# The Measure of Internal Rotation of the Knee in the Clinical Diagnosis of Association of Anterolateral Ligament and Anterior Cruciate Ligament Injury

**DOI:** 10.1055/s-0045-1811632

**Published:** 2025-11-04

**Authors:** Geraldo Luiz Schuck de Freitas, João Luiz Ellera Gomes

**Affiliations:** 1 Complexo Hospitalar da Santa Casa de Porto Alegre, Universidade Federal do Rio Grande do Sul, Porto Alegre, RS, Brazil; 2 Universidade Federal do Rio Grande do Sul, Porto Alegre, RS, Brazil; 3 Serviço de Ortopedia e Traumatologia do Hospital de Clínicas de Porto Alegre, Porto Alegre, RS, Brazil

**Keywords:** anterior cruciate ligament, diagnosis, knee joint, range of motion, articular, amplitude de movimento articular, articulação do joelho, diagnóstico, ligamento cruzado anterior

## Abstract

**Objective:**

To investigate the clinical correlation between internal knee rotation and the association of injuries between anterolateral (ALL) and anterior cruciate (ACL) ligaments.

**Methods:**

Thirty-eight knees of 19 fresh corpses (all males, mean age: 28-years-old) were evaluated by simulating physical examination through manual rotational tests at 90 degrees of flexion. Kirschner wires were placed in parallel in the femur and tibia, and measurements were obtained using a goniometer. The obtained data were compared against the intact ACL, then to progressive sections of the ACL, iliotibial tract, and anterolateral ligament.

**Results:**

Isolated release of the ACL induced an increase (+55.6%, *p* < 0.001) in internal rotation at 90 degrees of flexion, when compared to the the intact knee. After ACL release, associated release of the iliotibial tract (ITT) induced an increase (+31.6%, *p* < 0.001) in the internal rotation of the knee at 90 degrees of flexion, and a marked increase (+104%, *p* < 0.001) when compared to the ACL-intact knee. After ACL and ITT release, ALL release induced a significant increase (+27.8%, *p* < 0.001) in the internal rotation of the knee at 90 degrees of flexion, and in comparison with the intact ACL knee (+162%, *p* < 0.001).

**Conclusion:**

There is an increase in internal rotation of the knee in the ACL injury. The association with ALL injury leads to a pronounced increase of internal rotation when compared to the uninjured knee. Therefore, the presence of pronounced internal knee rotation is a clinical sign of associated injury to these structures.

## Resumo

**Objetivo:**

Investigar a correlação clínica entre a rotação interna do joelho e a associação de lesões nos ligamentos anterolateral (LAL) e cruzado anterior (LCA).

**Métodos:**

Trinta e oito joelhos de 19 cadáveres frescos (todos do sexo masculino, média de idade: 28 anos) foram avaliados simulando o exame físico por meio de testes manuais de rotação em 90 graus de flexão. Fios de Kirschner foram colocados paralelamente no fêmur e na tíbia e as medidas foram obtidas com um goniômetro. Os dados obtidos foram comparados com o LCA intacto e, em seguida, com secções progressivas do LCA, trato iliotibial (TIT) e LAL.

**Resultados:**

A secção isolada do LCA induziu um aumento (+55,6%, *p* < 0,001) na rotação interna em 90 graus de flexão em comparação a de o LCA intacto. Após a secção do LCA, a liberação associada do TIT induziu um aumento (+31,6%, *p* < 0,001) na rotação interna do joelho em 90 graus de flexão e um aumento acentuado (+104%, *p* < 0,001) em comparação ao joelho com LCA intacto. Após a secção do LCA e do TIT, a liberação do LAL induziu aumentos significativos (+27,8%, *p* < 0,001) na rotação interna do joelho em 90 graus de flexão e em comparação ao joelho com LCA intacto (+162%, *p* < 0,001).

**Conclusão:**

Há um aumento da rotação interna do joelho na lesão do LCA. A associação com a lesão do LAL causa um aumento pronunciado da rotação interna em comparação ao joelho íntegro. Portanto, a presença de rotação interna pronunciada do joelho é um sinal clínico de lesão associada dessas estruturas.

## Introduction

Studies on the existence of a distinct, ligamentous structure at the knee anterolateral aspect (ALL), 1 2 have once again sparked discussion on knee rotatory instability after an anterior cruciate ligament (ACL) injury. In injured knees, instability is evident through tibial anteriorization on the anteroposterior plane, along with enhanced knee internal rotation.

An important correlation between rotatory instability and anterolateral structures injuries has become evident. 3 However, it is still not clear which structure bears the most important role. Historically, knee surgeons have considered that tibial rotatory control is important to ensure knee stability. 4

To date, there is still no consensus on which procedure produces the best rotatory control for ACL reconstruction, however. 5 6 Recent studies of systematic revisions have concluded that, in some cases, the combination of intra- and extra-articular ACL reconstructions could improve rotatory instability. 7 8 Having a clinical sign that could improve the identification of the association between ACL with ALL injuries would help identify which patients would benefit from combined reconstruction.

This study aimed to determine the measurement of the knee internal rotation that could clearly demonstrate the association between ACL and ALL. More specifically, this investigation focused on the clinical assessment to aid surgeons in the diagnosis of these associated lesions. Our hypothesis was that an associated injury of anterolateral structures in an ACL-deficient knee could be clinically defined by the measurement of internal rotation.

## Materials and Methods

A total of 19 entire cadaveric specimens were chosen, comprising 38 knees with no evidence of ligament, chondral, or meniscal injury, with a minimum range of motion from 0 to 130 degrees. All cadavers were obtained from the local coroner's office, in accordance with the protocol approved by the Ethics Committee, under the CAAE: 45087815.0.0000.5327. The mean donor age was 28.42-years-old (range 18–47). All specimens were fresh, less than 18 hours from death, and none had been previously frozen.

### Surgical Approach

**Fig. 1:**
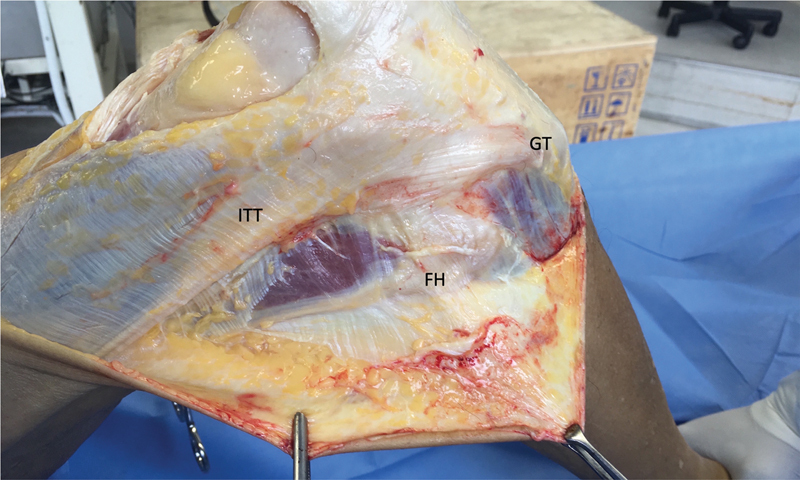
View of the lateral aspect of the right knee after removing the skin on the anterior and anterolateral surfaces of the knee, creating a large rectangular window. The Iliotibial tract (ITT) was then identified. **Abbreviations:** GT, Gerdy's tubercle; FH, fibular head.

A standardized protocol 9 for the ACL, iliotibial tract (ITT) and ALL of both knees was employed for dissection of 38 knees, from 19 fresh cadavers. Before the experiment, an adequate dissection of structures was performed, initiating the anatomical dissection through the removal of skin from the anterior and anterolateral aspects of the knee, creating a large rectangular window. The ITT was identified ( Fig. 1 ), and medial parapatellar arthrotomy proceeded with quadriceps tendon release to expose the intercondylar region and the ACL. The bias produced by cadaveric rigidity was avoided, since not all specimens had the same post mortem time.

The lower limb was positioned with the hip flexed at 45 degrees, the knee flexed at 90 degrees, and the foot flat on the table. After performing medial parapatellar arthrotomy to identify the femoral intercondylar region and structures of interest, before the experiment was started, all specimens were assessed to identify any injuries present. Next, with the knees positioned in 90 degrees of flexion, two parallel 2.0 Kirschner wires (K-wires) were inserted, one on the femoral intercondylar roof and the other into the anterior tibial tuberosity.

**Fig. 2:**
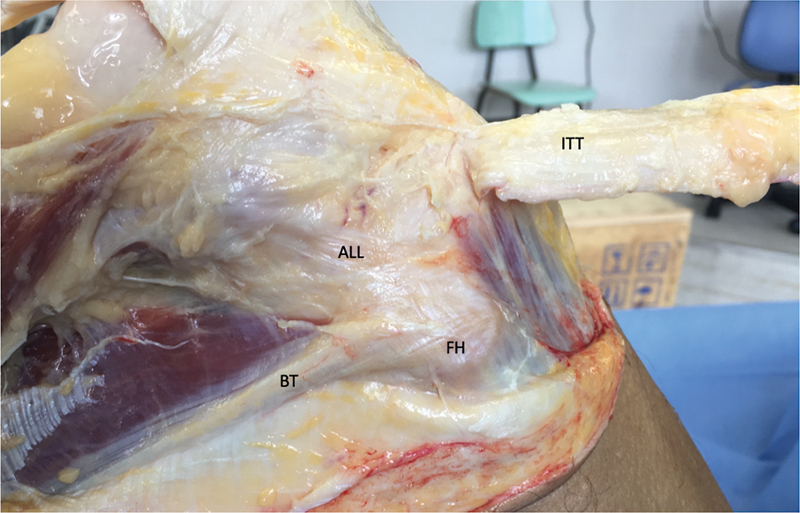
View of the lateral aspect of the right knee, the iliotibial tract (ITT) is dissected and reflected anteriorly to expose the anterolateral ligament (ALL). This way, we can access the ALL so we can identify it and then section it during the experiment. **Abbreviations:** FH, fibular head; BT, biceps tendon.

A lateral extension of the approach was performed, initiating over Gerdy's tubercle and extending proximally onto the thigh by 30 cm. A sequential release of the ACL and knee anterolateral structures was then performed, starting at the ITT and moving to the ALL, as determined in the experiment ( Fig. 2 ).

### Experiment

**Fig. 3:**
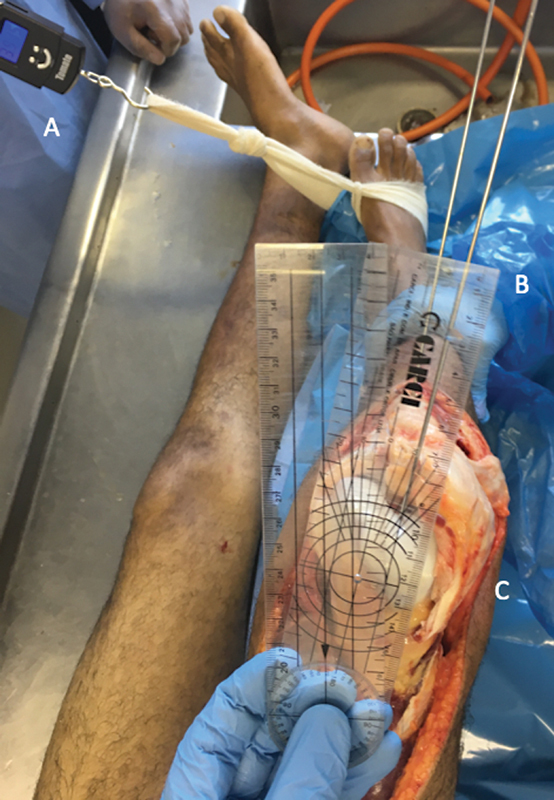
Anterior view of right knee showing the K-wires placed, ( **B** ) one on the roof of the femoral intercondylar and another placed on the anterior tibial tuberosity. Maximum internal rotation was ( **A** ) performed using a dynamometer and ( **C** ) measured with a goniometer.

A goniometer was employed to obtain kinematic data, measuring the angle formed between the two previously positioned K-wires. At the beginning of the experiment, before performing ligament release, we determined the maximum internal rotation of the tibia in relation to the fixed femur at 90 degrees of flexion. A STC-02 dynamometer (Mundial Comercio de Presentes Ltda.) was used to obtain a strength pattern during rotation. We applied maximum traction to the point that rotation was contained by ligament action on the normal knee, before releases were performed. Then, we recorded the dynamometer reading to determine the maximum force that could be applied to that specimen for the rest of the experiment. The rotation force was applied after every release, until the same value as the normal knee was obtained on the dynamometer. Assessment bias was controlled in such way ( Fig. 3 ). Measurements were taken as soon as the maximum rotation was attained.

**Fig. 4:**
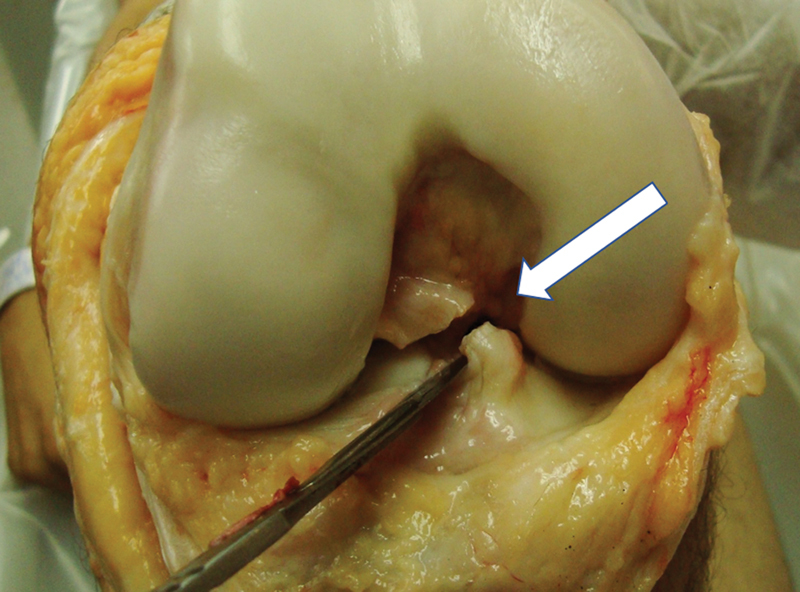
Anterior view of right knee in 90 degrees of flexion, the white arrow shows the section of the ACL with the scalpel. The internal rotation of the tibia was then measured.

With the knee maintained at 90 degrees, we reached maximum internal rotation and measured the angle between the two K-wires with a goniometer (PVC, 35 cm). Such data were collated and grouped as Intact Anterior Cruciate Ligament (INT ACL). Continuing with the experiment, the ACL was surgically cut ( Fig. 4 ), and the same measurements were performed and collected in a group named Injured Anterior Cruciate Ligament (INJ ACL).

In the next stage of the experiment, the ITT was surgically cut proximally and reflected inferiorly ( Fig. 2 ). The researchers took care to avoid disturbing the site of ITT tibial insertion.

**Fig. 5:**
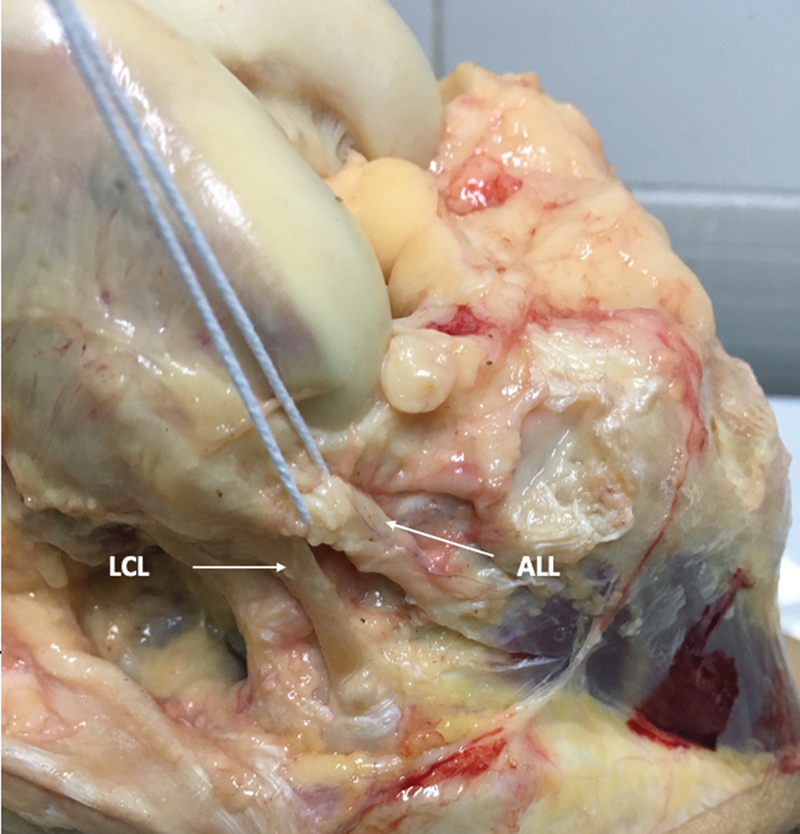
View of the lateral aspect of the right knee after dissection, anterior reflection, and removal of the ITT. The ALL can be individualized and dissected, separating it from the LCL. Next, we will cut the ALL and proceed to measure the internal rotation of the tibia.

Measurements were obtained with the same technique as described previously, and data were gathered in a group named Injured Anterior Cruciate Ligament associated to ITT release (INJ ACL + ITT). At this stage of the procedure, once the ITT was reflected, the ALL of the knee was dissected, applying a varus and internal rotation force at 30 and 60 degrees of flexion to elicit the effect of these structures under tension, such as ALL, described as a resistance to such motion, 4 as shown in Fig. 5 .

**Fig. 6:**
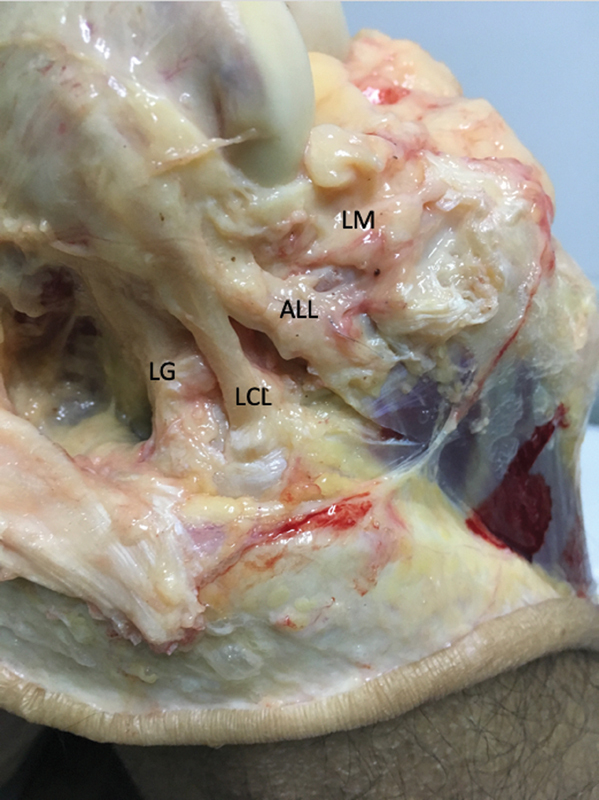
View of the lateral aspect of the right knee showing the lateral structures of the knee. From back to front, lateral gastrocnemius head (LG), lateral collateral ligament (LCL), anterolateral ligament (ALL), and lateral meniscus (LM). The popliteal tendon cannot be seen as they lie deep to LCL and ALL.

Once the area of interest containing the ALL had been exposed, the lateral collateral ligament (LCL) and the popliteus tendon (PT) were identified ( Fig. 6 ). The LCL was identified by palpation of its cylindrical structure at the site of distal attachment to the fibular head, just above the biceps femoris tendon attachment. Then, it was posteriorly exposed so that no tissue at the anterolateral aspect was ruptured. In order to confirm that no part of the LCL had been mistaken for any other additional structure, it was completely isolated from all other surrounding structures by following its fibers from distal to proximal with a blunt dissector. Under this ligament, the popliteus tendon was isolated and identified by traction of the popliteal fibular ligament.

After identification of the LCL and PT, the ALL was recognized and identified by tensioning its fibers and visualizing its femoral and tibial attachment sites. In the final stage of the experiment, a transverse surgically section of the ALL was performed, and the internal measurements were recorded, similar to previous stages of the experiment. Data were gathered in a group named Injured Anterior Cruciate Ligament, severed Iliotibial Tract, and severed Anterolateral Ligament (INJ ACL + ITT + ALL). The experimental procedure was the same for both knees.

### Statistical Analysis

Variables were described as the mean and standard deviation (SD). In order to compare the range of motion among procedures, analysis of variance (ANOVA) was applied for repeated measures, complemented by Bonferroni's post-hoc test where appropriate. The adopted level of significance was set at 5% ( *p* < 0.05), and analyses were performed with the IBM SPSS Statistics for Windows (IBM Corp.) software, version 21.0. A *t* test for paired samples was performed to compare mean internal rotation measurement values with the presence of ACL lesions and the addition of ALL sections. For this analysis, we adopted a significance level of 1% ( *p* < 0.001).

## Results

The knee ALL was identified as a distinguished anatomical structure in all 38 specimens, but only after the reflection of all ITT layers from its distal tibial attachment site. In all 38 knees, the ALL insertion to the lateral meniscus could be anatomically identified. Manipulation of the lateral meniscus in all directions showed that ALL attachment fibers headed in the same direction as the lateral meniscus when this structure was moved. Anterolateral ligament insertion over the tibia was, on average, halfway between the midpoint of Gerdy's tubercle and LCL insertion into the fibular head.

The isolated cut of the ACL induced a significant increase (+55.6%, *p* < 0.001) in internal rotation of the knee at 90 degrees of flexion when compared to the intact knee.

After ACL section, the association of ITT release induced a further increase of the knee internal rotation at 90 degrees of flexion (+31.6%, *p* < 0.001), and a very significant increase (+104%, *p* < 0.001) when compared to the intact knee.

After ACL and ITT section, the additional ALL release induced a significant increase in knee internal rotation at 90 degrees of flexion (+27.8%, *p* < 0.001), and when compared to the intact knee (+162%, *p* < 0.001).

A significant increase in knee internal rotation was observed as the ligament releases were performed. The specific section of the ACL led to a 55.6% increase in the mean internal rotation of the knee in relation to the femur when compared to the mean of the intact ACL group.

The addition of the ITT release increased the mean internal rotation of the knee by up to 102%. Finally, after the additional section of the ALL, the mean internal rotation of the knee increased even further, up to 162%.

There was a significant difference between the mean internal rotation measurements (21.13 ± 3.68) obtained for the isolated section of the ACL when compared to the mean internal rotation (35.57 ± 6.81) obtained when an ALL section was added ( Table 1 ). Therefore, this association created a 68.3% increase in internal rotation when compared to the measurements after the isolated section of the ACL ( Table 2 ).

**Table 1:** Rotational differences after serial section in 90 degrees of flexion

Range of motion	INT ACL	INJ ACL	INJ ACL +ITT	INJ ACL + ITT +ALL	*p* -value
	Mean ± SD	Mean ± SD	Mean ± SD	Mean ± SD	
Right knee internal rotation	13.6 ± 3.9	21.0 ± 3.8	27.4 ± 5.0	36.5 ± 7.4	< 0.001

**Abbreviations:** INJ ACL, injured anterior cruciate ligament; INJ ACL + ITT, injured anterior cruciate ligament and iliotibial tract; INJ ACL + ITT + ALL, injured anterior cruciate ligament, iliotibial, tract, and anterolateral ligament; INT ACL, intact anterior cruciate ligament; SD, standard deviation.

**Note:** The table indicate a statistical difference at a level of 5% by Bonferroni's post hoc test.

**Table 2:** Increased internal knee rotation after sequential ligament releases

Condition	Mean ± SD		Displacement (%)
INT ACL	13.57 ± 4.195		0
INJ ACL	21.13 ± 3.684		55.6
ACL + ITT	27.81 ± 4.543		104
ACL+ ITT + ALL	35.57 ± 6.812		162

**Abbreviations:** ACL + ITT, anterior cruciate ligament and iliotibial tract-associated injuries; ACL + ITT + ALL, anterior cruciate ligament, iliotibial tract, and anterolateral ligament-associated injuries; INJ ACL, isolated anterior cruciate ligament injury; INT ACL, intact anterior cruciate ligament; SD, standard deviation.

**Note:** Comparison of means, standard deviation and percentage of anterolateral displacement of internal rotation between groups.

The INJACL group (isolated ACL deficiency) had lower internal knee rotation ( *p* < 0.001) when compared with the ACL injury associated with the INJ ACL + ITT + ALL group.

This experiment shows a pronounced increase in the internal knee rotation, when the ALL injury was added to the ACL-deficient side, related to the release of the ALL.

## Discussion

Isolated ACL sections show a significant increase of the internal rotation of the knee at 90 degrees of flexion when compared to the intact knee. The associated ITT release on an ACL-deficient knee induced a further significant increase in the internal rotation of the knee at 90 degrees flexion when compared to an intact knee, highlighting its relevance. An additional ALL release significantly increased the knee internal rotation.

Our study demonstrated that there is a significant increase in knee internal rotation with ACL and ALL insufficiency and a severed ITT, suggesting that there is no specific structure that controls knee rotation. Nonetheless, our results demonstrate that ALL deficiency increases rotational instability when compared to the ACL and ITT sections. It is known that anterolateral structures are important restraints of knee internal rotation, 4 10 11 and act in synergy with the ACL. 12

The “clunk” produced on the pivot-shift test seems to have a weak correlation with the association of ACL and these structures injury. 13 Monaco et al. 13 were the first to hypothesize the relevance of the ALL among other anterolateral knee structures. A study by Parsons et al. 14 demonstrated that the contribution of the ALL increases significantly with increased knee flexion, while the ACL significantly reduces its contribution. The contribution of the ALL overcomes that of the ACL after 30 degrees of knee flexion. They concluded that the ALL is an important internal rotation stabilizer of the knee after 35 degrees of flexion. Sonnery-Cottet et al. 9 confirmed and stressed the involvement of the ALL in knee internal rotation control among the anterolateral structures.

In cases of ACL rupture, the rotational axis is displaced towards the medial compartment of the knee, increasing not only the anterior translation of the tibia over the femur, but also the internal rotation of the lateral compartment. 10 As a result, a significant increase in anterolateral structure recruitment is required to constrain such motion. The findings of our study confirm this, as seen in Table 1 . Insufficient postoperative rotatory control, seen after classic ACL reconstruction, could be caused by modification of the knee rotation center, but also due to the association with a lesion of its anterolateral structures. 15

A recent publication that reported the outcomes of ACL associated with ALL reconstruction with a follow-up lasting more than 2 years presented promising results in terms of clinical outcomes and rotational control. 16 Interestingly, in that series, the rate of contralateral ACL lesion (6.6%) was similar to that described in the literature, but the rate of graft rupture associated with ALL reconstruction (1.1%) was lower than previously published rates, 17 18 19 demonstrating that such an association may be extremely beneficial in some cases.

Regarding primary and secondary restraints, the failure of a primary restraint will lead to the recruitment of secondary structures to resist external forces and stabilize joint motion. In a study on the ALL, Dodds et al. 20 demonstrated that tibial internal rotation relative to the femur increases the distance between their insertions in that ligament, leading to tightening. The authors reported that the persistence of rotatory instability after ACL reconstruction could result from a failure to correct insufficiency of the anterolateral structures. Our study has shown that, in knees with ACL section (21.13 ± 4.19), that is, with simple anterolateral rotational instability, an additional ALL release (35.57 ± 6.81) significantly increased internal rotation ( *p* < 0.001).

This study has some limitations. The sequence of dissections might have over- or underestimated the individual stability of each component, due to interactions among those anatomical structures, which could not be assessed solely through the dissection technique. The measurement method is not electronically precise in data collection (such as navigation). However, all measurements were submitted to a rigid execution protocol and were always performed by the same researcher. We did not isolate or test Kaplan's fibers from the ITT, nor test the rotations through different force torques. Thus, our results may depend on the loads applied to those structures.

Despite meticulous dissection of the ITT, the ALL could have been injured, which would modify the results. We did not test an isolated section because this condition does not occur clinically. The association between ALL and LCA release was not assessed, as this is not yet possible. This is due to the impossibility of isolating the ALL in order to perform its release, without first retracting the ITT strictly following the dissection technique, as was widely discussed by Sonnery-Cottet et al. 9

## Conclusion

The ACL injury increases the knee's internal rotation. There is a demonstrated correlation between the ACL and ALL injury, leading to a pronounced increase in internal rotation when compared to the uninjured knee (above 100%). Therefore, the presence of pronounced internal rotation of the knee is a clinical sign of associated injury to these structures, which can assist surgeons in the diagnosis and guide appropriate treatments.
